# Generation of human induced pluripotent stem cell lines from a fetus with congenital long QT syndrome and her healthy parents

**DOI:** 10.1016/j.scr.2025.103848

**Published:** 2025-09-30

**Authors:** Manesha Putra, Bettina F. Cuneo, Congwu Chi, Zhen Zhang, Linnea Prudell, Kunhua Song

**Affiliations:** a Division of Maternal Fetal Medicine, Department of Obstetrics and Gynecology, University of Colorado School of Medicine, Aurora, CO 80045, USA; b Departments of Surgery and Pediatrics, University of Arizona College of Medicine, Tucson, AZ 85724 USA; c Department of Internal Medicine, University of South Florida Morsani College of Medicine, Tampa, FL 33602, USA; d Heart Institute, Center for Regenerative Medicine, University of South Florida Morsani College of Medicine, Tampa, FL 33602, USA; e Heart Institute, Human Stem Cell Core Facility, University of South Florida Morsani College of Medicine, Tampa, FL 33602, USA

## Abstract

Long QT syndrome (LQTS) is a channelopathy that predisposes affected individuals to ventricular arrhythmias and cardiac arrest. Here, a human induced pluripotent stem cell (hiPSC) line was generated from amniotic fluid cells (AFCs) of a 32-week fetus diagnosed with LQTS. Additionally, two iPSC lines were generated from peripheral blood mononuclear cells (PBMCs) of the fetus’s healthy biological parents. Genome sequencing revealed that the fetus with LQTS carried a *de novo KCNH2* variant, c.1898A > G (p.Asn633Ser). All three iPSC lines demonstrated normal morphology, karyotyping, and pluripotency. These iPSC lines provide a valuable *in vitro* model for LQTS caused by *KCNH2* mutations.

## 1. Resource utility

A healthy 32-year old woman in her second pregnancy presented at 32 weeks with fetal 2° atrioventricular block, ventricular tachycardia and a fetal heart rate <3rd for gestational age (Putra et al., 2023). There was no family history of inherited arrhythmias and parental ECGs were normal. The assumed diagnosis of fetal long QT syndrome (LQTS) was confirmed by amniocentesis, which revealed a *de novo KCNH2* variant, c.1898A > G (p.Asn633Ser). HiPSC lines generated from amniocytes of this fetus and blood cells from her healthy parents could serve as an invaluable resource for *in vitro* disease modeling of congenital LQTS, candidate drug testing, and the development of personalized therapeutic plans.

## 2. Resource details

Long QT syndrome (LQTS) is an inherited channelopathy that occurs in 1/2000 individuals (Schwartz et al.,2009). Pathogenic variants in at least 15 genes encoding ion channel proteins in cardiomyocytes have been associated with LQTS (Nakano & Shimizu, 2016). The most common types of LQTS are due to pathogenic variants in the genes KCNQ1 (LQT1), KCNH2 (LQT2), and SCN5A (LQT3). Pathogenic variants in the LQTS genes result in a prolonged repolarization (QT interval), predisposing affected individuals to polymorphic ventricular tachycardia and sudden death before and after birth (Crotti et al.,2013; Schwartz, 2004). De novo pathogenic KCNH2 and SCN5A variants are more likely to manifest the signature LQTS rhythm of *torsades de pointes* and have a high mortality risk than familial LQTS (Cuneo et al., 2013; Strand et al., 2020). Mutations in the *KCNH2* gene, which encodes hERG1, a voltage-gated potassium channel protein, account for 35–40 % of the diagnosed LQTS cases (Curran et al.,1995; Sanguinetti et al., 1995). As the genetic etiologies for unexplained stillbirth increase (Merc et al., 2024), patient-specific human induced pluripotent stem cells (hiPSCs) that can be differentiated into various cardiac lineage cells and used for cardiovascular disease modelling become more important (Sallam et al., 2014). Cardiomyocytes derived from hiPSCs (hiPSC-CMs) are emerging as a powerful system to study inherited LQTS caused by *KCNH2* variants (Feng et al., 2021; Jain et al., 2023; Ukachukwu et al., 2022). Therefore, these three hiPSC lines generated in this study present a valuable platform for modelling LQTS pathogenesis and testing potential therapeutics. The clinical utility of this platform includes functionally assessing variants of uncertain pathogenicity and serving as an *in vitro* tool to determine the most effective pharmacotherapy for individuals.

**Fig. 1.**
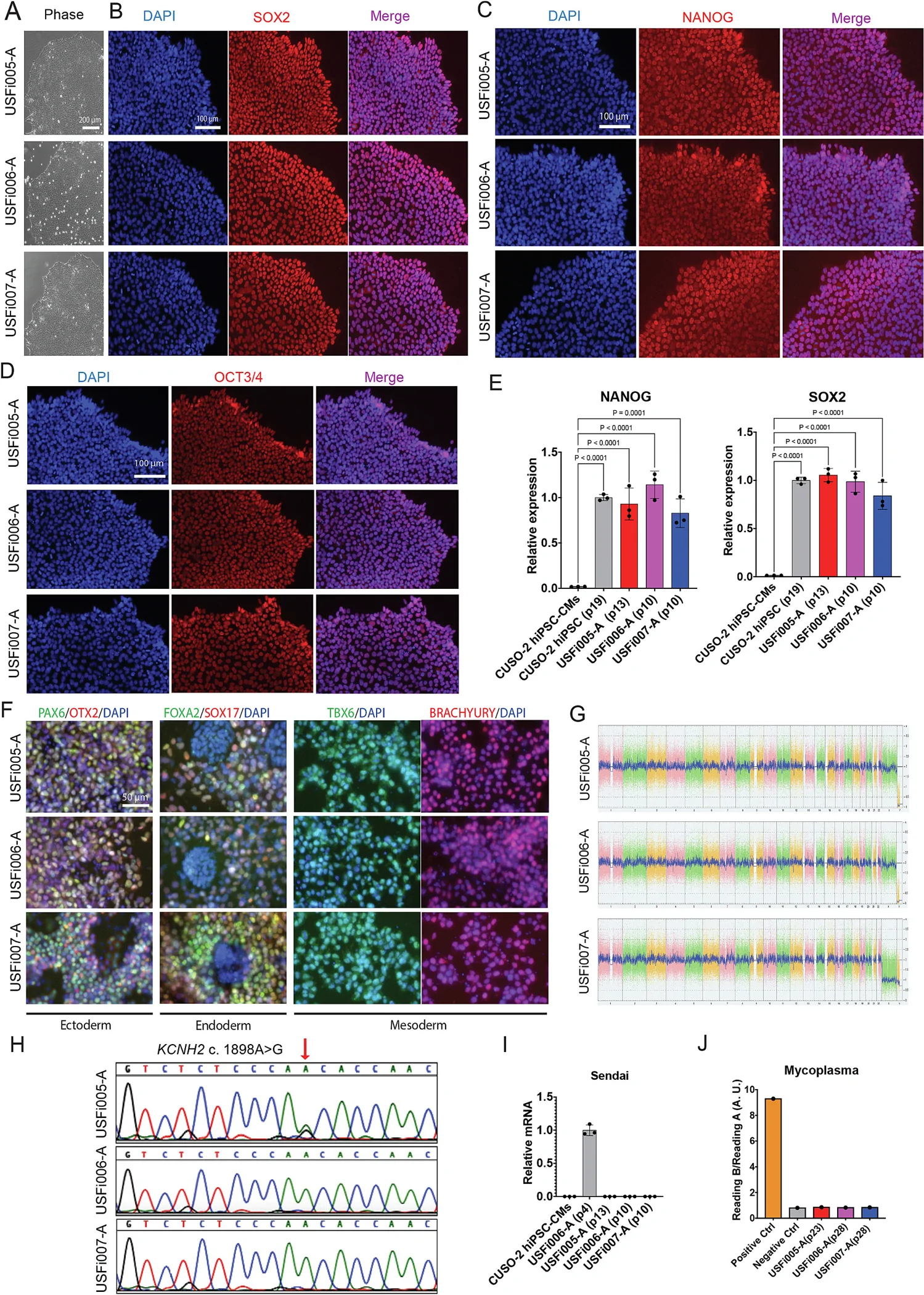
Characterization of WT hiPSC lines, and a patient-derived hiPSC line with c.1898A > G mutation in *KCNH2*. (A–D) Phase microscopy images and immunocytochemistry of SOX2, NANOG, and OCT3/4 (in red) in the indicated hiPSC lines. Scale bars, 100 μm. (E) Quantitative PCR (qPCR) analysis of NANOG and SOX2 expression in the indicated cells. Each filled circle represents one independent experiment. Data are presented as mean ± SD. *P* values were calculated using one-way ANOVA with Turkey’s multiple comparisons test. (F) Fluorescence immunocytochemistry of lineage markers in derivatives differentiated from the indicated hiPSC lines. Scale bar, 50 μm. (G) Karyotype images of iPSC lines. (H) Sanger sequencing revealed a heterozygous mutation in the *KCNH2* gene. Genomic DNA was extracted from the indicated hiPSC lines. (I) qPCR analysis of Sendai viral vectors was performed in the indicated cells, with three replicates for each line. (J) Mycoplasma contamination in the indicated cells was examined using the MycoAlert Mycoplasma Detection Kit, with one assay for each. (For interpretation of the references to colour in this figure legend, the reader is referred to the web version of this article.)

In this study, we derived one hiPSC line from a fetus with congenital LQTS carrying a heterozygous c.1898A > G mutation in *KCNH2* gene (USFi005-A). Two additional hiPSCs lines were derived from healthy biological parents of the fetus, including a 32-year-old female (USFi006-A) and a 34-year-old male (USFi007-A). Whole-genome sequencing demonstrated that the fetus carries c.1898A > G mutation, while both parents carry wild-type (WT) alleles of the *KCNH2* gene. Amniotic fluid cells (AFCs) were collected from the fetus, and peripheral blood mononuclear cells (PBMCs) were obtained from her parents during a clinical visit. Somatic cell reprogramming was performed using non-integrated Sendai virus driven expression of human Yamanaka factors (OCT3/4, SOX2, KLF4, c-MYC). All three hiPSC lines exhibited typical stem cell morphology (Fig. 1A, Table 1) and expressed SOX2, NANOG, and OCT-3/4 pluripotency markers (Fig. 1B–D). The expression of these pluripotency markers was further quantified by reverse transcription-quantitative polymerase chain reaction (RT-qPCR). All three hiPSC lines showed comparable expression levels of NANOG and SOX2 to a control hiPSC line (CUSO-2) used in multiple previous studies (Fig. 1E) (Chi et al., 2019; Knight et al., 2021). Furthermore, all three iPSC lines can be differentiated into all three germ layers-endoderm, ectoderm, and mesoderm, demonstrating their pluripotency (Fig. 1F).

Cytogenetic analysis revealed USFi005-A and USFi006-A have normal 46XX karyotype, while USFi007-A has a 46XY karyotype (Fig. 1G). Sanger sequencing confirmed that the heterozygous *KCNH2* mutation was presented only in USFi005-A, and not in USFi006-A or USFi007-A (Fig. 1H). Short Tandem Repeat (STR) analysis results verified that each iPSC line generated matched the identity of its respective somatic donor cell. Additionally, qPCR analysis demonstrated that expression of the non-integrating Sendai virus was present at low passage numbers (e.g., USFi006-A, p4), while absent at high passage numbers (e.g., USFi005-A, p13; USFi006-A, p10; and USFi007-A, p10) (Fig. 1I). Three hiPSC lines were negative for mycoplasma (Fig. 1J).

## 3. Materials and Methods

### 3.1. Cell culture and reprogramming

PBMCs were isolated from peripheral blood samples using Ficoll gradient separation (Sigma #GE17–5442-03). The enriched PBMCs were expanded and reprogrammed to hiPSCs according to the instruction of the Erythroid Progenitor Reprogramming Kit (STEMCELL Technologies #05924). Briefly, PBMCs were expanded in Erythroid Expansion Medium for 7 days before reprogramming. Reprogramming was initiated by infecting 2×10^5^ cells with Sendai virus carrying human Yamanaka factors, provided in CytoTune-iPS 2.0 Sendai Reprogramming Kit (Invitrogen #A16517) on Matrigel-coated plate. On day 2, 3 and 5 after infection, 1 ml of ReproTeSR medium was added to cells. On day 7, the culture medium was replaced with fresh ReproTeSR medium, and the medium was refreshed daily until hiPSC colonies appeared.

AFCs were first cultured in AmnioMAX-II Complete Medium (ThermoFisher Scientific #11269016) for 4–5 days to allow cell attachment. Non-attached cells were removed, and the medium was replaced every 3–5 days. AFCs were passaged when reached 70–80 % confluence by using 0.05 % trypsin and seeded at a split ratio of 1:4–1:6. For reprogramming, 2.5 × 10^4^ AFCs were seeded on Matrigel-coated plate 2 days before transduction. Transduction was performed by using the CytoTune-iPS 2.0 Sendai Reprogramming Kit. The medium was replaced with fresh AmnioMAX-II Complete Medium every other day up to 7 days post-infection. On day 7, AFCs were transferred to MatriGel (Corning #354277) – coated plate with TeSR-E7 medium (STEMCELL Technologies #05914) supplemented with 10 μM Y-27632 (ApexBio #501012282). Starting on day 8, the culture medium was replaced with fresh TeSR-E7 medium daily until hiPSC colonies appeared.

hiPSCs were cultured in mTeSR1 (STEMCELL Technologies #85850) on plates coated with MatriGel. mTeSR1 was refreshed daily. 10 μM Y-27632 was added to the medium for 24 h after passage. hiPSCs were routinely passed with Gentle Cell Dissociation Reagent (STEMCELL Technologies #1000485) at 1:3 ratio every 4–6 days. All cells were maintained in a 37 °C humidified incubator with 5 % CO_2_.

### 3.2. Immunofluorescence staining

Immunofluorescence staining was performed as previously described (Chi et al., 2023). The primary and secondary antibodies used in this study are listed in Table 2.

### 3.3. RT-qPCR

Total RNA was extracted from cells using TRIzol reagent (Invitrogen #15596026) according to the manufacturer’s instructions. cDNA synthesis was performed using the iScript Reverse Transcription Supermix (Biorad #1708840). Quantitative PCR (qPCR) was carried out using SYBR PowerUP master mix (Applied Biosystems #4309155) on the StepOne Real-Time PCR System (Applied Biosystems) with gene specific primers (Table 2). The 18S ribosomal RNA gene was used as an internal control.

### 3.4. Trilineage differentiation

Endoderm, ectoderm, and mesoderm differentiation was induced from hiPSCs using STEMdiff Trilineage Differentiation Kit (STEMCELL Technologies #05230). Differentiation was performed at passage 27 (USFi005-A), passage 22 (USFi006-A), and passage 22 (USFi007-A).

### 3.5. Karyotyping

Karyotypic analysis of patient derived hiPSC lines, USFi005-A at passage 26 (p26), USFi006-A at passage 21 (p21), and USFi007-A at passage 21 (p21), was performed using the KaryoStat + Assay (Thermo Fisher Scientific).

### 3.6. Sanger sequencing

Genomic DNA from hiPSCs were prepared as crude lysates by using hot 50 mM NaOH and then neutralized with 1 M Tris-HCl. The genomic region of the *KCNH2* gene was amplified by PCR using primers flanking c.1898A > G mutation (Table 2) and the Phusion high-fidelity PCR Kit (New England Biolabs #E0553S). PCR products were sequenced by MCLAB.

### 3.7. STR analysis

Frozen cell pellets (1 × 10^6^ cells) from hiPSCs as well as their respective somatic parental cells (AFCs for USFi005-A, PBMCs for USFi006-A and USFi007-A) were collected. STR analysis was performed using the CellCheck Cell Line Authentication service by IDEXX BioAnalytics.

### 3.8. Mycoplasma detection

Cell culture supernatant was collected by spinning the cell sample at 200×*g* for 5 min. Mycoplasma contamination was evaluated using the MycoAlert Assay Control Set (Lonza #LT07–518) and the MycoAlert Mycoplasma Detection Kit (Lonza #LT07–418).

## Supplementary Material

1

## Figures and Tables

**Table 1:** Characterization and validation.

Classification	Test	Result	Data

**Morphology**	Photography Bright field	Normal	Fig. 1A
	Quantitative analysis	RT-qPCR confirmed comparable expression levels of NANOG and SOX2 to a control iPSC line (CUSO-2).	Fig. 1E
**Genotype**	Karyotype (KaryoStat + Assay) and resolution	46XX for both USFi005-A and USFi006-A, 46XY for USFi007-A; Resolution at 1 Mb	Fig. 1G
**Identity**	Microsatellite PCR (mPCR) OR	Not performed	N/A
	STR analysis	16 loci tested, 100 % identical	Available with the authors.
**Mutation analysis (IF APPLICABLE)**	Sequencing	USFi005-A: heterozygous c.1898A > G USFi006-A and USFi007-A: wild-type	Fig. 1H
	Southern Blot OR WGS	Not performed	
**Microbiology and virology**	Mycoplasma	Mycoplasma testing by luminescence: Negative	Fig. 1J
**Differentiation potential**	Directed differentiation	Verified expression of markers for all three germ layers	Fig. 1F
**List of recommended germ layer markers**	**Expression of these markers has to be demonstrated at mRNA (RT PCR) or protein (IF) levels, at least 2 markers need to be shown per germ layer**	Ectoderm: PAX6, OTX2 Endoderm: SOX17, FOXA2 Mesoderm: BRACHYURY, TBX6	Fig. 1F
**Donor screening (OPTIONAL)**	HIV 1 + 2 Hepatitis B, Hepatitis C	Not performed	Not performed
**Genotype additional info (OPTIONAL)**	Blood group genotyping	Not performed	Not performed
	HLA tissue typing	Not performed	Not performed

**Table 2:** Reagents details.

	Antibodies used for immunocytochemistry/flow-cytometry			
	Antibody	Dilution	Company Cat #	RRID

Pluripotency Marker	Mouse anti-SOX2	1:100	Santa Cruz Biotechnology Cat# sc-365823	AB_10842165
Pluripotency Marker	Rabbit anti-Nanog	1:400	Cell Signaling Technology Cat# 3580 s	AB_2150399
Pluripotency Marker	Mouse anti-OCT3/4	1:100	Santa Cruz Biotechnology Cat# sc-5279	AB_62805
Differentiation Marker (Ectoderm)	Rabbit anti-PAX6	1:200	Thermo Fisher Scientific Cat# 42–6600	AB_2533534
Differentiation Marker (Ectoderm)	Mouse anti-OTX2	1:200	Thermo Fisher Scientific Cat# MA5–15854	AB_11155193
Differentiation Marker (Endoderm)	Rabbit anti-FOXA2	1:100	Thermo Fisher Scientific Cat# 701698	AB_2576439
Differentiation Marker (Endoderm)	Mouse anti-SOX17	1:100	Thermo Fisher Scientific Cat# MA5–24885	AB_2725396
Differentiation Marker (Mesoderm)	Rabbit anti-TBX6	1:200	Thermo Fisher Scientific Cat# PA5–35102	AB_2552412
Differentiation Marker (Mesoderm)	Rabbit anti-BRACHYURY	1:1000	Cell Signaling Technology Cat# 81694	AB_2799983
Secondary Antibody	Alexa Fluor 488 Goat Anti-Rabbit IgG (H + L)	1:800	Thermo Fisher Scientific Cat# A-11034	AB_2576217
Secondary Antibody	Alexa Fluor 555 Goat Anti-Rabbit IgG (H + L)	1:800	Thermo Fisher Scientific Cat# A-21428	AB_2535849
Secondary Antibody	Alexa Fluor 555 Goat Anti-Mouse IgG (H + L)	1:800	Thermo Fisher Scientific Cat# A-21422	AB_2535844
	Primers			
	Target	Size of band	Forward/Reverse primer (5'–3')	

Genotyping	KCNH2 (c.1898A > G)	305 bp	CGTGCTGTTCTTGCTCATGT/CACAGCCAATGAGCATGACG	
Pluripotency Marker (RT-qPCR)	NANOG		CTCCAACATCCTGAACCTCAGC/CGTCACACCATTGCTATTCTTCG	
Pluripotency Marker (RT-qPCR)	SOX2		GCTACAGCATGATGCAGGACCA/TCTGCGAGCTGGTCATGGAGTT	
Housekeeping gene (RT-qPCR)	18S ribosomal RNA		GCCGCTAGAGGTGAAATTCTTA/CTTTCGCTCTGGTCCGTCTT	
Sendai virus plasmid (RT-qPCR)	Sendai virus genome	181 bp	GGATCACTAGGTGATATCGAGC/ACCAGACAAGAGTTTAAGAGATATGTATC	

**Resource Table:** 

Unique stem cell lines identifier	USFi005-A USFi006-A USFi007-A
Alternative name(s) of stem cell lines	CUUSFi001-A/USFi005-A/LQT1 CUUSFi002-A/USFi006-A/LQT2 CUUSFi003-A/USFi007-A/LQT3
Institution	University of Colorado School of Medicine and University of South Florida Morsani College of Medicine
Contact information of distributor	Kunhua Song, ksong34@usf.edu
Type of cell lines	iPSC
Origin	Human
Additional origin info required	Age: 0 (USFi005-A), 32 (USFi006-A) and 34 (USFi007-A) Sex: Female (fetus/USFi005-A), female (mother/USFi006-A) and male (father/ USFi007-A) *Ethnicity if known: White*
Cell Source	AFCs (USFi005-A), PBMCs (USFi006-A, USFi007-A)
Clonality	Clonal
Method of reprogramming	Nonintegrating Sendai virus expression of human KLF4, OCT3/4, SOX2, and c-MYC
Genetic Modification	Yes
Type of Genetic Modification	Spontaneous mutation
Evidence of the reprogramming transgene loss (including genomic copy if applicable)	RT/qPCR
Associated disease	Long QT syndrome
Gene/locus	*KCNH2* (7q35-q36) USFi005-A: heterozygous *KCNH2* (c.1898A > G) USFi006-A: wild-type *KCNH2* USFi007-A: wild-type *KCNH2*
Date archived/stock date	USFi005-A: 5/23/2023 USFi006-A: 6/27/2023 USFi007-A: 6/26/2023
Cell line repository/bank	https://hpscreg.eu/cell-line/USFi005-A, https://hpscreg.eu/cell-line/USFi006-A, https://hpscreg.eu/cell-line/USFi007-A.
Ethical approval	The work was approved by the University of Colorado Institutional Review Board (IRB) under Protocol #22–2310 “iPS-cardiomyocyte cells and pharmacological testing in a fetus with long QT syndrome”

## Data Availability

Data will be made available on request.

## Appendix A. Supplementary data

Supplementary data to this article can be found online at https://doi.org/10.1016/j.scr.2025.103848.

## References

[R1] Chi C, Knight WE, Riching AS, Zhang Z, Tatavosian R, Zhuang Y, Moldovan R, Rachubinski AL, Gao D, Xu H, Espinosa JM, Song K, 2023. Interferon hyperactivity impairs cardiogenesis in down syndrome via downregulation of canonical Wnt signaling. iScience 26, 107012. 37360690 10.1016/j.isci.2023.107012 PMC10285545

[R2] Chi C, Leonard A, Knight WE, Beussman KM, Zhao Y, Cao Y, Londono P, Aune E, Trembley MA, Small EM, Jeong MY, Walker LA, Xu H, Sniadecki NJ, Taylor MR, Buttrick PM, Song K, 2019. LAMP-2B regulates human cardiomyocyte function by mediating autophagosome-lysosome fusion. PNAS 116, 556–565. 30584088 10.1073/pnas.1808618116 PMC6329949

[R3] Crotti L, Tester DJ, White WM, Bartos DC, Insolia R, Besana A, Kunic JD, Will ML, Velasco EJ, Bair JJ, Ghidoni A, Cetin I, Van Dyke DL, Wick MJ, Brost B, Delisle BP, Facchinetti F, George AL, Schwartz PJ, Ackerman MJ, 2013. Long QT syndrome and intrauterine fetal death. J. Am. Med. Assoc. 309, 1473–1482. 10.1001/jama.2013.3219 PMC3852902 23571586

[R4] Cuneo BF, Etheridge SP, Horigome H, Sallee D, Moon-Grady A, Weng HY, Ackerman MJ, Benson DW, 2013. Arrhythmia phenotype during fetal life suggests long-QT syndrome genotype: risk stratification of perinatal long-QT syndrome. Circ. Arrhythm. Electrophysiol. 6, 946–951. 23995044 10.1161/CIRCEP.113.000618 PMC4151528

[R5] Curran ME, Splawski I, Timothy KW, Vincent GM, Green ED, Keating MT, 1995. A molecular basis for cardiac arrhythmia: HERG mutations cause long QT syndrome. Cell 80, 795–803. 7889573 10.1016/0092-8674(95)90358-5

[R6] Feng L, Zhang J, Lee C, Kim G, Liu F, Petersen AJ, Lim E, Anderson CL, Orland KM, Robertson GA, Eckhardt LL, January CT, Kamp TJ, 2021. Long QT syndrome KCNH2 variant induces hERG1a/1b subunit imbalance in patient-specific induced pluripotent stem cell-derived cardiomyocytes. Circ. Arrhythm. Electrophysiol. 14, e009343. 33729832 10.1161/CIRCEP.120.009343 PMC8058932

[R7] Jain A, Stack O, Ghodrati S, Sanchez-Conde FG, Ukachukwu CU, Salwi S, Jimenez-Vazquez EN, Jones DK, 2023. KCNH2 encodes a nuclear-targeted polypeptide that mediates hERG1 channel gating and expression. PNAS 120, e2214700120. 36626562 10.1073/pnas.2214700120 PMC9934303

[R8] Knight WE, Cao Y, Lin YH, Chi C, Bai B, Sparagna GC, Zhao Y, Du Y, Londono P, Reisz JA, Brown BC, Taylor MRG, Ambardekar AV, Cleveland JC Jr., McKinsey TA, Jeong MY, Walker LA, Woulfe KC, D’Alessandro A, Chatfield KC, Xu H, Bristow MR, Buttrick PM, Song K, 2021. Maturation of pluripotent stem cell-derived cardiomyocytes enables modeling of human hypertrophic cardiomyopathy. Stem Cell Rep. 16, 519–533. 10.1016/j.stemcr.2021.01.018 PMC7940251 33636116

[R9] Merc MD, Kotnik U, Peterlin B, Lovrecic L, 2024. Further exploration of cardiac channelopathy and cardiomyopathy genes in stillbirth. Prenat. Diagn. 44, 1062–1072. 38813989 10.1002/pd.6616

[R10] Nakano Y, Shimizu W, 2016. Genetics of long-QT syndrome. J. Hum. Genet. 61, 51–55. 26108145 10.1038/jhg.2015.74

[R11] Putra M, Lee YM, Bucholz E, Ross EL, Galan H, Behrendt N, Micke K, Chow FS, Zaretsky MV, Cuneo BF, 2023. Successful management of Fetal Torsades de Pointes and long QT syndrome by a cardio-obstetrical team. JACC Case Rep. 27, 102110. 38094730 10.1016/j.jaccas.2023.102110 PMC10715977

[R12] Sallam K, Kodo K, Wu JC, 2014. Modeling inherited cardiac disorders. Circ J. 78, 784–794. 24632794 10.1253/circj.cj-14-0182 PMC6530482

[R13] Sanguinetti MC, Jiang C, Curran ME, Keating MT, 1995. A mechanistic link between an inherited and an acquired cardiac arrhythmia: HERG encodes the IKr potassium channel. Cell 81, 299–307. 7736582 10.1016/0092-8674(95)90340-2

[R14] Schwartz PJ, 2004. Stillbirths, sudden infant deaths, and long-QT syndrome: puzzle or mosaic, the pieces of the jigsaw are being fitted together. Circulation 109, 2930–2932. 15210606 10.1161/01.CIR.0000133180.77213.43

[R15] Schwartz PJ, Stramba-Badiale M, Crotti L, Pedrazzini M, Besana A, Bosi G, Gabbarini F, Goulene K, Insolia R, Mannarino S, Mosca F, Nespoli L, Rimini A, Rosati E, Salice P, Spazzolini C, 2009. Prevalence of the congenital long-QT syndrome. Circulation 120, 1761–1767. 19841298 10.1161/CIRCULATIONAHA.109.863209 PMC2784143

[R16] Strand S, Strasburger JF, Cuneo BF, Wakai RT, 2020. Complex and novel arrhythmias precede stillbirth in fetuses with de novo long QT syndrome. Circ. Arrhythm. Electrophysiol. 13, e008082. 32421437 10.1161/CIRCEP.119.008082 PMC7241276

[R17] Ukachukwu CU, Jimenez-Vazquez EN, Jain A, Jones DK, 2022. hERG1 channel subunit composition mediates proton inhibition of rapid delayed rectifier potassium current (IKr) in cardiomyocytes derived from hiPSCs. J. Biol. Chem. 299, 102778. 36496073 10.1016/j.jbc.2022.102778 PMC9867984

