# Supplementary material for: Generation of human induced pluripotent stem cell lines from a fetus with congenital long QT syndrome and her healthy parents
Source: Stem Cell Res. Author manuscript; Available in PMC 2026 Aug 23. (PMC13499978; doi:10.1016/j.scr.2025.103848)
Supplement: Supplementary file 1 [file NIHMS2183844-supplement-1.pdf]

Thermo Fisher SCIENTIFIC

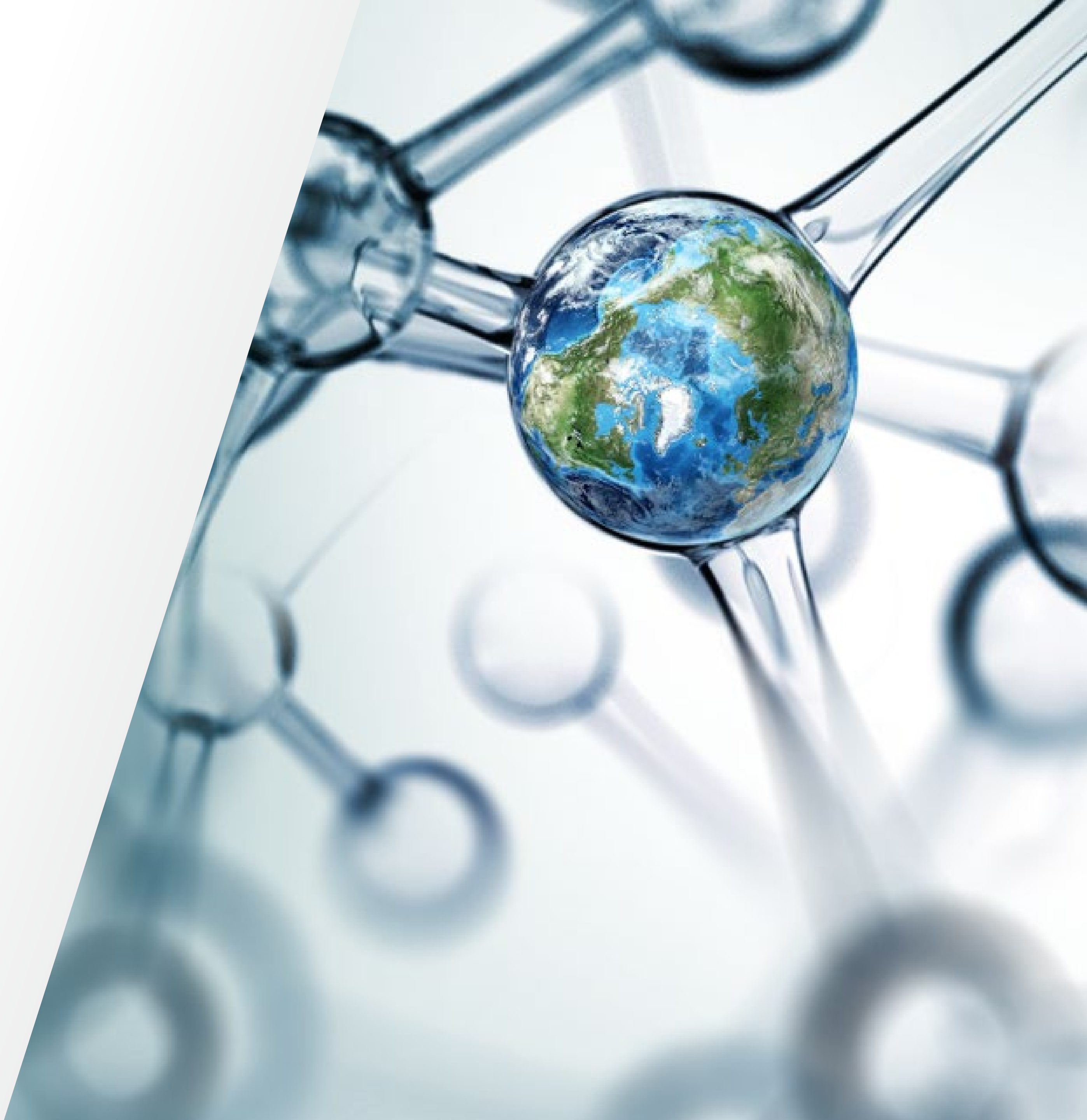

KaryoStat+ ^{TM} Assay Report

Client Name: **University of South Florida**
Quote No: **13238472 SO**
Date: **29-Jan-2025**
Prepared by: **Kevin J. Velez**
Reviewed by: **Meera Kumar**

The world leader in serving science

Assay background

- Traditionally, genetic stability of pluripotent stem cells has been confirmed via g-banded karyotyping, a method performed by professional cytogeneticists. This method requires provision of log-phase cells by the researcher. Chromosomal spreads from 20 individual cells are stained and visually inspected for aberrations, and images captured under a microscope for subsequent reporting.
- Recently, researchers have adopted alternative methods employing molecular, array-based approaches to help reduce subjectivity and streamline sample submission. Conveniently, frozen cell pellets are collected instead of live cells. Resolution is often slightly higher (1Mb or less) with these assays than with traditional karyotyping (5-10 Mb). The limit of detection for percent of cells containing aberrations is lower, however, since it is a population-based assay rather than evaluating one cell at a time.
- KaryoStat+ Assay services represents one such alternative to g-banded karyotyping for **all non-transformed human cells**. This includes primary cells, stem cells, and *in vitro*-differentiated cells. The assay offers accurate genotyping (cell ID) plus whole-genome coverage for accurate detection of chromosomal abnormalities with a typical turnaround time of 3-4 weeks. This assay is **not recommended for transformed or immortalized cell lines** due to the large number of chromosomal rearrangements observed. These cell lines are unlikely to pass internal QC requirements and therefore provide inconclusive results.
- We welcome you to find out more about the services at the following site:

https://www.thermofisher.com/us/en/home/life-science/stem-cell-research/stem-cell-services/karyostat-karyotyping-service.html

Summary of Services

Project Summary:

- University of South Florida is interested in services provided by the Life Technologies Corporation in the analysis of four (4) client-provided samples using the KaryoStat+ assay.

Service Description:

- The KaryoStat™ assay allows for digital visualization of chromosome aberrations with a resolution similar to g-banding karyotyping. The size of structural aberration that can be detected is > 2 Mb for chromosomal gains and > 1 Mb for chromosomal losses (the resolution depends on the location of the aberration in the chromosome. Due to a lower probe density on the telomere ends and centromeres, the resolution in those locations may be closer to 5Mb). The KaryoStat array is optimized for balanced whole-genome coverage with a low-resolution DNA copy number analysis, the assay covers all 36,000 RefSeq genes, including 14,000 OMIM® targets. The assay enables the detection of aneuploidies, submicroscopic aberrations, and mosaic events.

Methods:

1. Genomic DNA purification:
   - Cells were prepared according to the PureLink™ Genomic DNA Purification Kit (Catalog #: K1820-02) and quantified using the NanoDrop ONE^C (Catalog # : 701-058108).

2. GeneArray® Preparation:
   - 100 ng total gDNA was used to prepare the GeneArray® for KaryoStat according to the manual, and is an array that looks for SNPs, copy number variants and single nucleotide polymorphisms across the genome.

Sample Information

#	Sample ID	Status
KS-24440	LQT1	Complete
KS-24441	LQT2	Complete
KS-24442	LQT3	Complete
KS-24443	FF	Complete

Table 1: Karyostat⁺ Sample ID. Customer provided sample information

KaryoStat+ Results: KS-24440

1. KaryoStat+ analysis of this sample revealed that it originated from a female individual.
2. No chromosomal aberrations were found when comparing against the reference dataset.

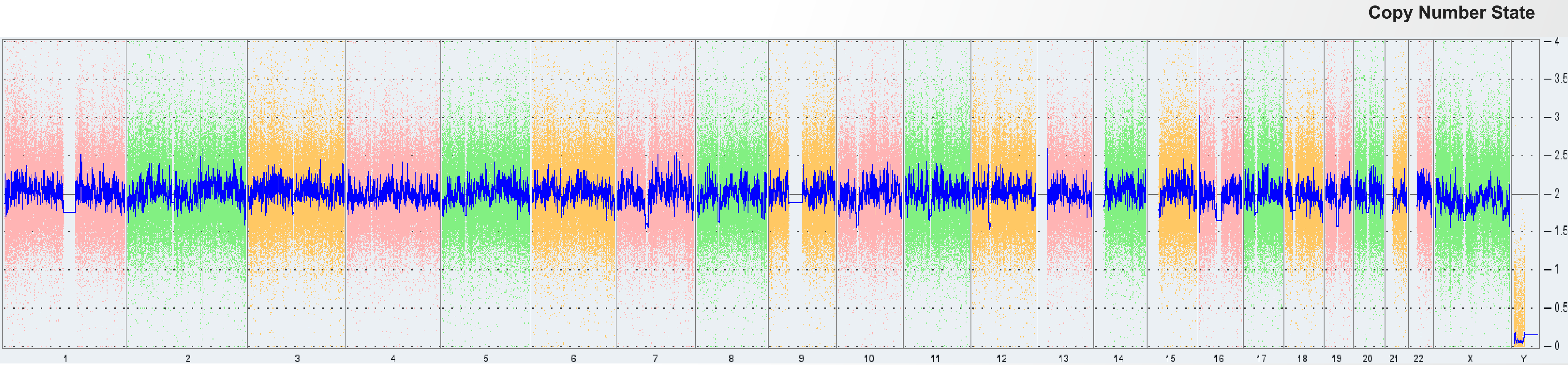

Figure 1: Whole genome view. The whole genome view displays all somatic and sex chromosomes in one frame with high level copy number. The smooth signal plot (right y-axis) is the smoothing of the log2 ratios which depict the signal intensities of probes on the microarray. A value of 2 represents a normal copy number state (CN = 2). A value of 3 represents chromosomal gain (CN = 3). A value of 1 represents a chromosomal loss (CN = 1). The pink, green and yellow colors indicate the raw signal for each individual chromosome probe, while the blue signal represents the normalized probe signal which is used to identify copy number and aberrations (if any). Aberrations when present are indicated by red arrows.

Disclaimer: This assay was conducted solely for the listed investigator/institution. The results of this assay are for research use only.

KaryoStat+ Results: KS-24441

1. KaryoStat+ analysis of this sample revealed that it originated from a female individual.
2. No chromosomal aberrations were found when comparing against the reference dataset.

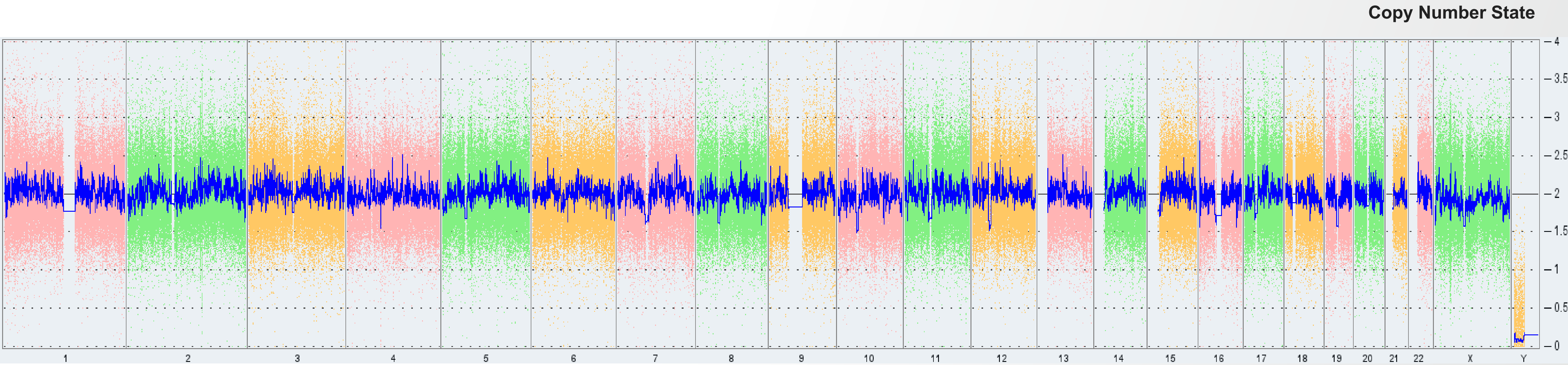

Figure 2: Whole genome view. The whole genome view displays all somatic and sex chromosomes in one frame with high level copy number. The smooth signal plot (right y-axis) is the smoothing of the log2 ratios which depict the signal intensities of probes on the microarray. A value of 2 represents a normal copy number state (CN = 2). A value of 3 represents chromosomal gain (CN = 3). A value of 1 represents a chromosomal loss (CN = 1). The pink, green and yellow colors indicate the raw signal for each individual chromosome probe, while the blue signal represents the normalized probe signal which is used to identify copy number and aberrations (if any). Aberrations when present are indicated by red arrows.

Disclaimer: This assay was conducted solely for the listed investigator/institution. The results of this assay are for research use only.

KaryoStat+ Results: KS-24442

1. KaryoStat+ analysis of this sample revealed that it originated from a male individual.
2. No chromosomal aberrations were found when comparing against the reference dataset.

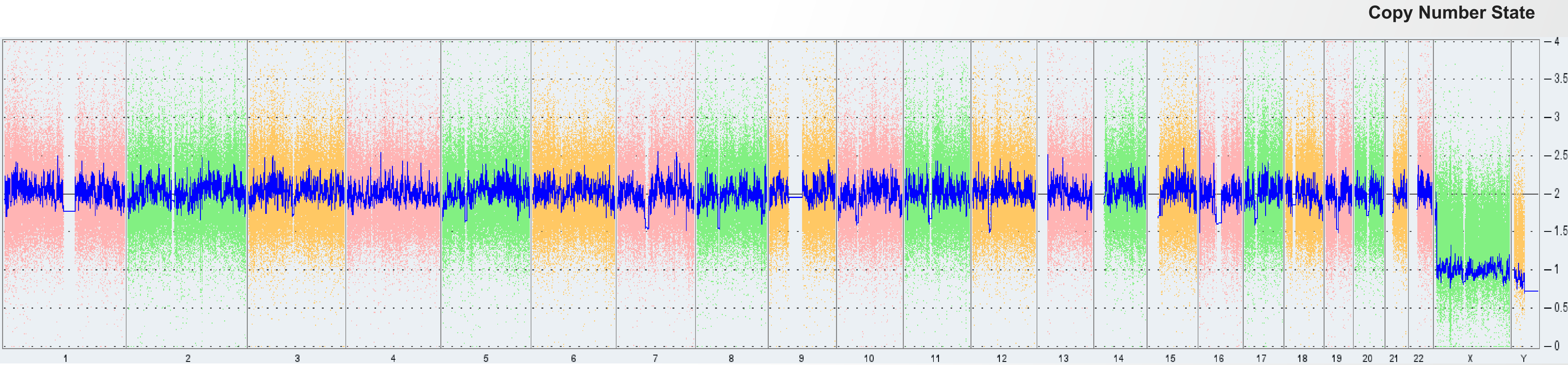

Figure 3: Whole genome view. The whole genome view displays all somatic and sex chromosomes in one frame with high level copy number. The smooth signal plot (right y-axis) is the smoothing of the log2 ratios which depict the signal intensities of probes on the microarray. A value of 2 represents a normal copy number state (CN = 2). A value of 3 represents chromosomal gain (CN = 3). A value of 1 represents a chromosomal loss (CN = 1). The pink, green and yellow colors indicate the raw signal for each individual chromosome probe, while the blue signal represents the normalized probe signal which is used to identify copy number and aberrations (if any). Aberrations when present are indicated by red arrows.

Disclaimer: This assay was conducted solely for the listed investigator/institution. The results of this assay are for research use only.

KaryoStat+ Results: KS-24443

1. KaryoStat+ analysis of this sample revealed that it originated from a female individual.
2. No chromosomal aberrations were found when comparing against the reference dataset.

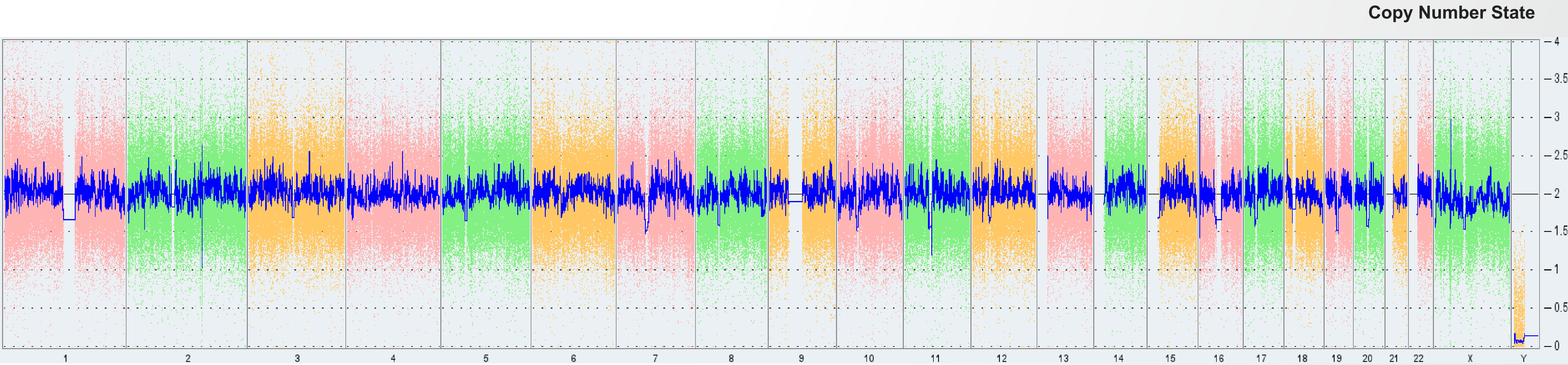

Figure 4: Whole genome view. The whole genome view displays all somatic and sex chromosomes in one frame with high level copy number. The smooth signal plot (right y-axis) is the smoothing of the log2 ratios which depict the signal intensities of probes on the microarray. A value of 2 represents a normal copy number state (CN = 2). A value of 3 represents chromosomal gain (CN = 3). A value of 1 represents a chromosomal loss (CN = 1). The pink, green and yellow colors indicate the raw signal for each individual chromosome probe, while the blue signal represents the normalized probe signal which is used to identify copy number and aberrations (if any). Aberrations when present are indicated by red arrows.

Disclaimer: This assay was conducted solely for the listed investigator/institution. The results of this assay are for research use only.

Appendix

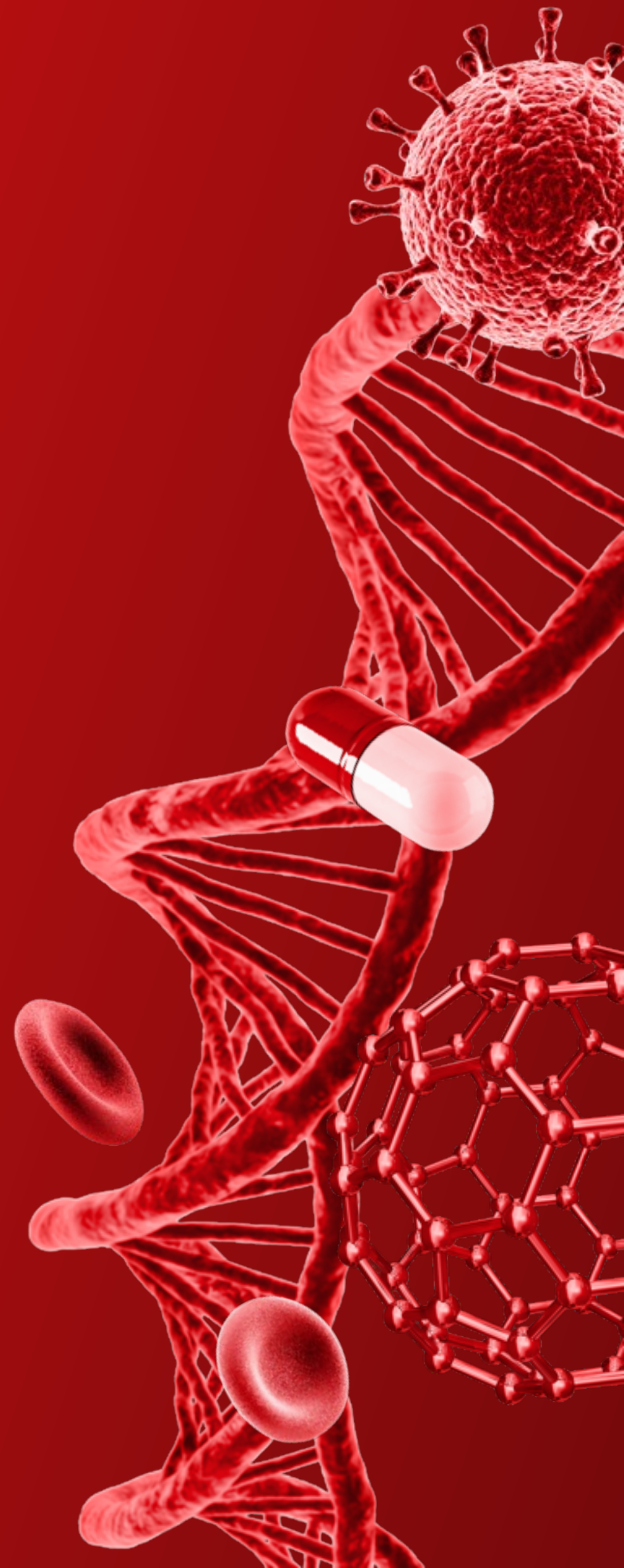

KaryoStat+ Results Navigation (This is an example)

ThermoFisher SCIENTIFIC

Chromosome	Type	Cytoband Start	CN State	Size (kbp)
1	GainMosaic	q24.2	2.45	79,196
18	Loss	q21.33	1	16,724

This table summarizes any aberrations that were identified

Table 2. KaryoStat+ analysis. Chromosomal aberrations are indicated in the table shown. See supplemental data for more details.

The Whole Genome View below displays the microarray data, visualized as smoothed copy number state for each region of the genome, calculated from probe signal intensity. Gains show a higher copy number, losses show a lower copy number. A fractional copy number state indicates mosaicism.

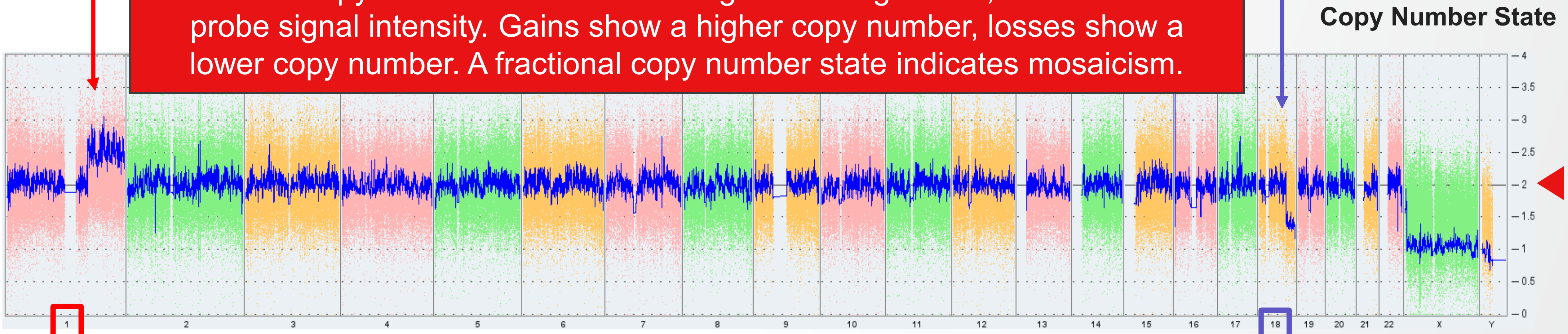

Expected copy number is two for all but the sex chromosomes for samples derived from male cells

Figure 1: Whole genome view. The whole genome view displays all somatic and sex chromosomes in one frame with high level copy number. The smooth signal plot (right y-axis) is the smoothing of the log2 ratios which depict the signal intensities of probes on the microarray. A value of 2 represents a normal copy number state (CN = 2). A value of 3 represents chromosomal gain (CN = 3). A value of 1 represents a chromosomal loss (CN = 1). The pink, green and yellow colors indicate the raw signal for each individual chromosome probe, while the blue signal represents the normalized probe signal which is used to identify copy number and aberrations (if any). The whole genome view analysis revealed chromosomal aberrations indicated by the red arrow. Aberrations close to the detection limit of the Karyostat assay requires further investigation for confirmation of its presence.*

Disclaimer: This assay was conducted solely for the listed investigator/institution. The results of this assay are for research use only.

Supplemental KaryoView is only provided if aberrations are detected (This is an example)

Aberrations Table

Chromosome	Type	Cytoband Start	CN State	Size (kbp)
1	GainMosaic	q24.2	2.45	79,196
18	Loss	q21.33	1	16,724

Chromosome in which the aberration was detected

Type of aberration detected

Cytoband Start: location where the aberration was detected

CN State: Copy Number state

Size of the aberration in kilobase pairs

KaryoView with Genes Affected

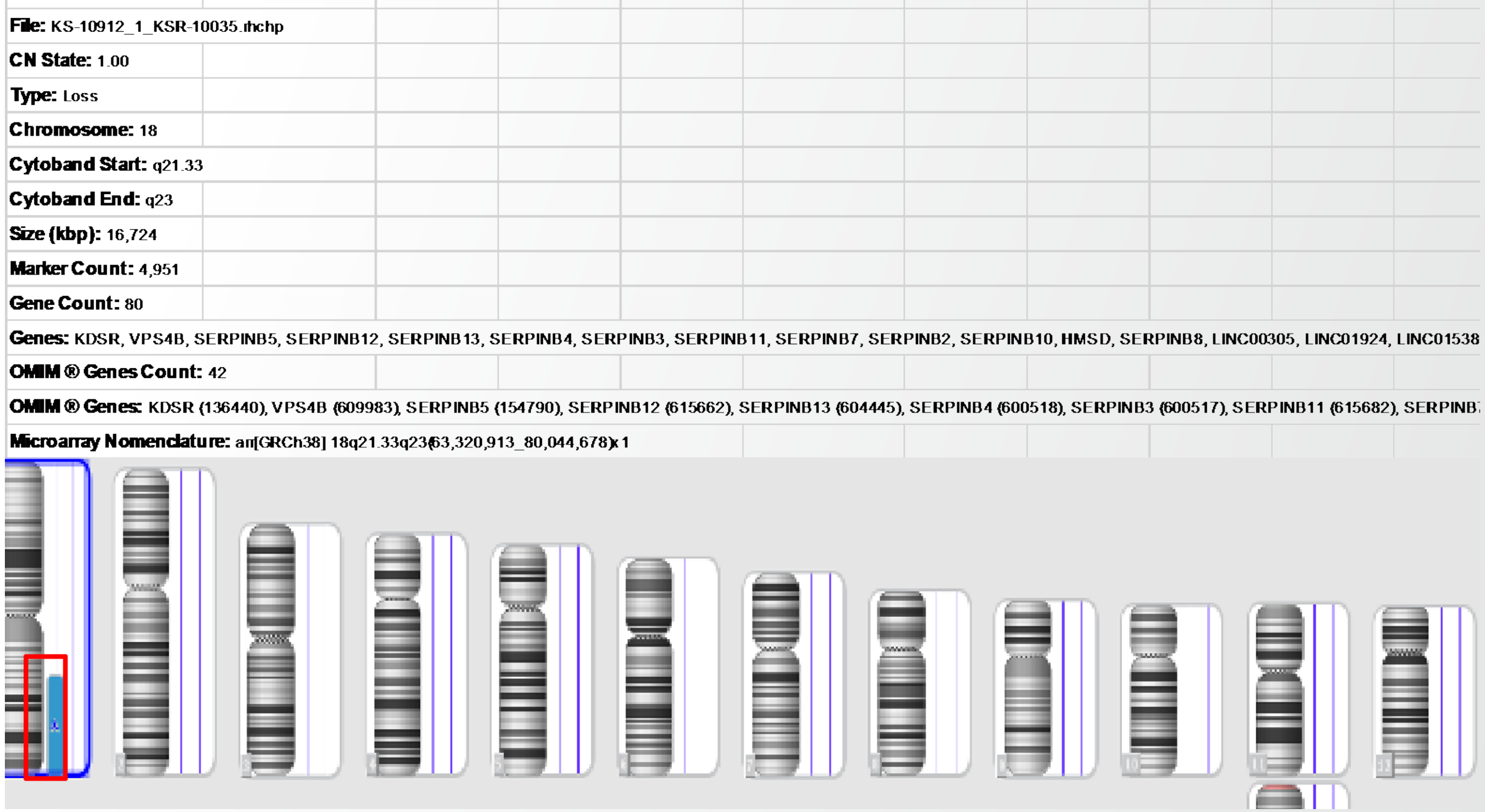

KaryoStat+ Assay Services Frequently Asked Questions (1 of 6)

- **What citations have referenced this assay?**
  - *Answer: As of April 2022, nearly 100 publications have cited KaryoStat assays. Review the latest list on Google Scholar here.*
- **How many cells are required to run one experiment?**
  - *We require a cell pellet of at least 2 million cells per sample.*
- **Can I have a copy of our raw KaryoStat+ data?**
  - *No, the only additional data that can be provided is the genotype data, available as a text file on a case-by-case basis. Karyostat raw data undergoes a significant amount of processing before turned into the presented data, which requires expertise not easily gained. In addition, raw KaryoStat+ assay data files are very large (>500 MB) and are thus not easily shared. Submit an inquiry to CUBCharacterization@thermofisher.com for more details.*
- **Why don't I get a chromosome map if no aberrations are detected?**
  - *Answer: KaryoStat+ is different from traditional G-banding in that it is a molecular assay. No chromosomal staining is performed and, therefore, no images of your cells' chromosomes are generated. A convenient chromosome visualization tool (KaryoView) is employed to illustrate the locations of aberrations when they are detected. If none are detected, a KaryoView is not generated by the software.*
- **What is the difference between Karyostat and Karyostat+ assays?**
  - *The Karyostat+ assay leverages a new array system (Cytoscan HT-CMA, SKU 906025) to allow integration of automation into array processing. Both assays leverage SNP probes to look at chromosomal aberrations. Karyostat has about ~150k probes, Karyostat+ has about 1.1M probes. The higher probe density provides more accurate results and allows a higher resolution down to 1 Mb.*

KaryoStat+ Assay Services Frequently Asked Questions (2 of 6)

- **My G-banding report says my cell has a normal karyotype, but some aberrations were reported when analyzed in the KaryoStat+ service. Why do the results differ?**
  - *G-banding has a typical resolution of 5-10 Mb and cannot detect submicroscopic gains/losses. The KaryoStat+ microarray has a resolution of 1 Mb. Due to the higher resolution of the Karyostat+ microarray, some abnormalities that are below the limit of detection of the g-banding analysis may be detected.*
- **Can KaryoStat+ pick up clonal differences within a line or due to accidental contamination of cell lines, similar to g-banding?**
  - *KaryoStat+ is run as a pooled DNA analysis. If non-clonal lines were submitted, differences can be picked up with a detection limit of 30%, anything below 30% will not be picked up reliably.*
- **My report states that several aberrations were found. What does this mean for the biology of my cells?**
  - *For all aberrations found, we provide detailed chromosome location and the genes that are affected. From this data alone, we cannot predict how these genes may affect the biology of the cells.*
- **Are the KaryoStat+ reports certified by a cytogeneticist?**
  - *We currently do not offer cytogenetic certification of the KaryoStat+ reports, and they are intended for research use only.*
- ***May I submit DNA instead of a cell pellet?***
  - *We can only accept frozen cell pellets, so that we can control the DNA quality for a successful assay.*

KaryoStat+ Assay Services Frequently Asked Questions (3 of 6)

Thermo Fisher SCIENTIFIC

- **Which microarray is used in the Karyostat+ service?**
  - *We use the Cytoscan HT-CMA 96F array system (SKU: 906025) in the service that has a resolution of approximately 1 Mb.*
- **Can I run samples using the higher resolution KaryoStat HD microarray?**
  - *No, this assay is currently not available. KaryoStat+ detects chromosomal gains at a resolution twice that of KaryoStat.*
- **I want to perform the KaryoStat+ AND PluriTest services. How many cells do I need to send?**
  - *Since we are performing two different extractions (i.e. DNA for KaryoStat+ and RNA for PluriTest), we need a separate aliquot for each service (i.e. two aliquots of 2M cells each)*
- **Are volume discounts available?**
  - *Yes, you may receive volume discounts based upon the number of samples submitted simultaneously for analysis. Further discounts can be applied with yearly volume targets.*
- **May I get a discount if ordering karyotyping and pluripotency testing at the same time?**
  - *The KaryoStat+ and PluriTest services employ two independent workflows, so no operational efficiencies are gained by running samples at the same time. Discounts are available for submitting multiple samples to the same service or by committing to yearly volume targets.*
- **How is the microarray data normalized?**
  - *There are two steps involved in the workflow. The first step involves normalizing the pixel intensities of samples collected from the scanner to control oligos hybridized to the corner of the array. This process called gridding, converts the Dat file into a Cel file. The second step involves adjusting the copy number of the samples to the reference set in the software. The $\log_2$ ratio is the log2 of the ratio of signal intensity of a sample probe set to that of the reference total intensity for the same probe set. The blue trace seen on the whole genome view represents the normalized signal.*

KaryoStat+ Assay Services Frequently Asked Questions (4 of 6)

- **Why do I have a peak in the whole genome view?**
  - *It may be a gain or loss that doesn't meet the 1000 kb limit of detection or it's a false positive and requires further investigation.*

- **We have some feeder-dependent iPSC lines that need to be tested for karyotype. Could we send the cell pellet cultured on feeders or do we need to provide feeder-free samples?**
  - *The probes on the microarray are for human cells and theoretically expected to not cross -react with mouse cells. However, many of the genes are conserved between the two species and cross-reactions cannot be completely ruled out. We do recommend samples with limited (no more than 10%) to no MEFs. Samples with MEFs can still be processed but we cannot guarantee successful completion of the assay.*

- **What is the merits and drawbacks of KaryoStat™ in comparison with the G -banding? In addition, in G-banding karyotyping, we usually analyze 20 cells. How many cells does KaryoStat analyze?**
  - *KaryoStat will not be able to detect inversions, balanced translocations and SNVs. In terms of the number of cells that are analyzed, KaryoStat is run as a pooled DNA analysis. If non -clonal lines are submitted, differences can be picked up with a detection limit of 30%. G-banding looks at individual cells, which allows clonal populations to be picked up with a detection limit of 5%. The low cell number screened in g-banding however may misrepresent the presence or distribution of aberrations*

- **Can aberrations <5MB be detected with g-banded karyotyping?**
  - *G-banding typically has a resolution of 5 Mb or greater for detection. Therefore, any aberration < 5MB will likely not be detected with with g-banding. KaryoStat + allows an unbiased detection of aberrations down to 1 Mb due to the molecular approach*

- **I see a tendency of outliers in certain loci on the whole genome view (WGV). Is this occurrence cell line specific, cell type specific and/or analysis specific?**
  - *Generally, these tendencies do not seem to correlate with samples indicating that these signal variations are in the range of experimental noise. While consistent appearance of aberrant signal within the same cell background may indicate genome alterations, the KaryoStat+ assay has a detection limit of 1MB for chromosomal gains and 1Mb for losses and aberrations and is not developed to detect below that threshold. Therefore, aberrations with sizes around or below that limit will not be called consistently (especially at the telomere ends) and should thus not be considered reliable.*

KaryoStat+ Assay Services Frequently Asked Questions (5 of 6)

ThermoFisher SCIENTIFIC

- **What is the limit of detection for KaryoStat+ Assay?**
  - *Gains: > 1 Mb*
  - *Losses: > 1 Mb*
  - *Note that the resolution for a specific sample will depend on the location of the aberration in the chromosome. Due to a lower probe density on the telomere ends and centromeres, the resolution in those locations may be closer to 5 Mb.*

- **Can KaryoStat+ assay pick up clonal differences within a line or due to accidental contamination of cell lines, similar to g-banding?**
  - *Karyostat™ is run as a pooled DNA analysis. If non-clonal lines were submitted, differences can be picked up with a detection limit of 30%, anything below 30% will not be picked up reliably. G -banding looks at individual cells which allows clonal populations to be picked up with a detection limit of 5%. The low sample number (generally only 20-40 cells are screened) however makes G-banding based clonal assessments very unreliable.*

- **My sample has not passed the KaryoStat+ assay after multiple attempts. What's the explanation for this?**
  - *There are various reasons why samples do not pass. Below are possible reasons for failure:*
    - *Presence of feeders of different species (for example MEF for hiPSC)*
    - *High number of chromosomal abnormalities (for example transformed cell lines or cancer cells)*
    - *Low cell number (less than 2M)*
    - *Poorly dissociated samples (overdigestion of cells with dissociation buffers will impact DNA quality)*

  - *We recommend that new samples be prepared following our guidelines for a re-run.*

KaryoStat+ Assay Services Frequently Asked Questions (6 of 6)

Thermo Fisher SCIENTIFIC

- **Sample X had a chromosomal aberration that as detected by KaryoStat. Do you know what percentage of the cells contains the non-mosaic gain/loss? Was it 100% of the cells?**
  - *Aberrations that are not mosaic means every cell has it, thus 100% of the cells have the aberration.*

- **Why is there no raw signal for some regions? For example, there is an empty region in the middle of chromosome 1. What do the empty regions mean?**
  - *The empty region represents the centromeres of the chromosomes (there are no SNP probes in those regions) and in some cases also represents the telomeres, which on some chromosomes have also have lower probe coverage.*

- **Should I be concerned that a small subset of the raw signals show a copy number of 1? For example, at the beginning of chromosome 1, although the normalized blue signal (blue line) indicates a copy number of ~1.5, there are some raw signals (pink dots) that appear near copy number 1.**
  - *The telomere ends tend to have a lower SNP probe density, and this may cause a little more variation in the signals that you see and explains why the copy number state may go down a bit. Our algorithms take this into account when aberration calls are made, thus there is no need to be concerned.*

- **I want to publish my KaryoStat+ Assay results in a peer-reviewed journal article. How do I represent my data and cite the service?**
  - *Congratulations on preparation of your manuscript. We're excited to have played a small supporting role in your work. Suggested verbiage for your Materials & Methods section: "Genomic stability of cells was evaluated using the KaryoStat+ Assay service provided by Thermo Fisher Scientific. No aberrations were detected."*

Cell ID Assay Services Frequently Asked Questions (1 of 2)

Thermo Fisher SCIENTIFIC

- **How does Cell Identification work?**
  - *Cell Identification service leverages the SNP data that is collected during the KaryoStat™ + assay. The KaryoStat™ + assay allows for digital visualization of chromosome aberrations with 5x higher resolution than g-banding karyotyping by relying on SNP probes across the human genome. This underlying SNP data enables DNA fingerprint matching of human cell lines through correlation analysis of unique SNP signatures between two or more samples.*

- **What correlations are used to determine DNA matching between samples?**
  - *The correlation between a sample and itself has a value of 100. Samples with correlations of over 95% are considered having an identical genetic background (color coded Green in results table). Samples with correlations of less than 95% contained different genetic backgrounds (color coded Red in results table).*

- **Do I have to submit my reference cell line with each run?**
  - *To establish a reference file, the cell sample must be submitted and designated as a reference cell line on the Characterization Sample Submission form. After that, Cell ID analysis against this designated reference file can be requested for future service runs. See Data Retention Policy below. If a reference cell sample is not designated for each run, a comparison between the submitted samples for that run will be performed.*
  - *Data Retention Policy: Thermo Fisher Scientific will store the reference file for up to 5 years. We will retain data from up to 20 reference samples per customer. All other sample files not designated as a reference file will be discarded after 1 year.*

Cell ID Assay Services Frequently Asked Questions (2 of 2)

Thermo Fisher SCIENTIFIC

- **I want to compare samples run with KaryoStat+Cell ID to samples that were previously analyzed using KaryoStat Cell ID. Do I need to pay to have my previous sample re-processed with KaryoStat + Cell ID?**
  - *You will not need to pay for previous KaryoStat Cell ID samples that were originally designated as "reference cell line" to be re-analyzed on KaryoStat + Cell ID. Please inform your PMO partner with the sample name, project number and a new cell pellet to reprocess a sample at no additional cost.*

- **What are the options for reference samples for Cell ID runs?**
  - *There are three options, depending on where the reference sample is coming from:*

    Option 1: Compare submitted samples to each other *- all samples submitted are compared against each other.*

    Option 2: Compare submitted sample to Cell ID reference sample *- In this option your sample will be compared to a reference sample designated at the time of submission or to a sample submitted previously as a reference.*

    Option 3: Create a reference sample *– Use this option to designate a sample as reference at the time of submission of samples. References can be used in the same run or saved for later use. Thermo Fisher Scientific will store the reference file for up to 5 years. We will retain data from up to 20 reference samples per customer.*

For additional questions please contact : cellanalysis.support@thermofisher.com

Thermo Fisher
SCIENTIFIC

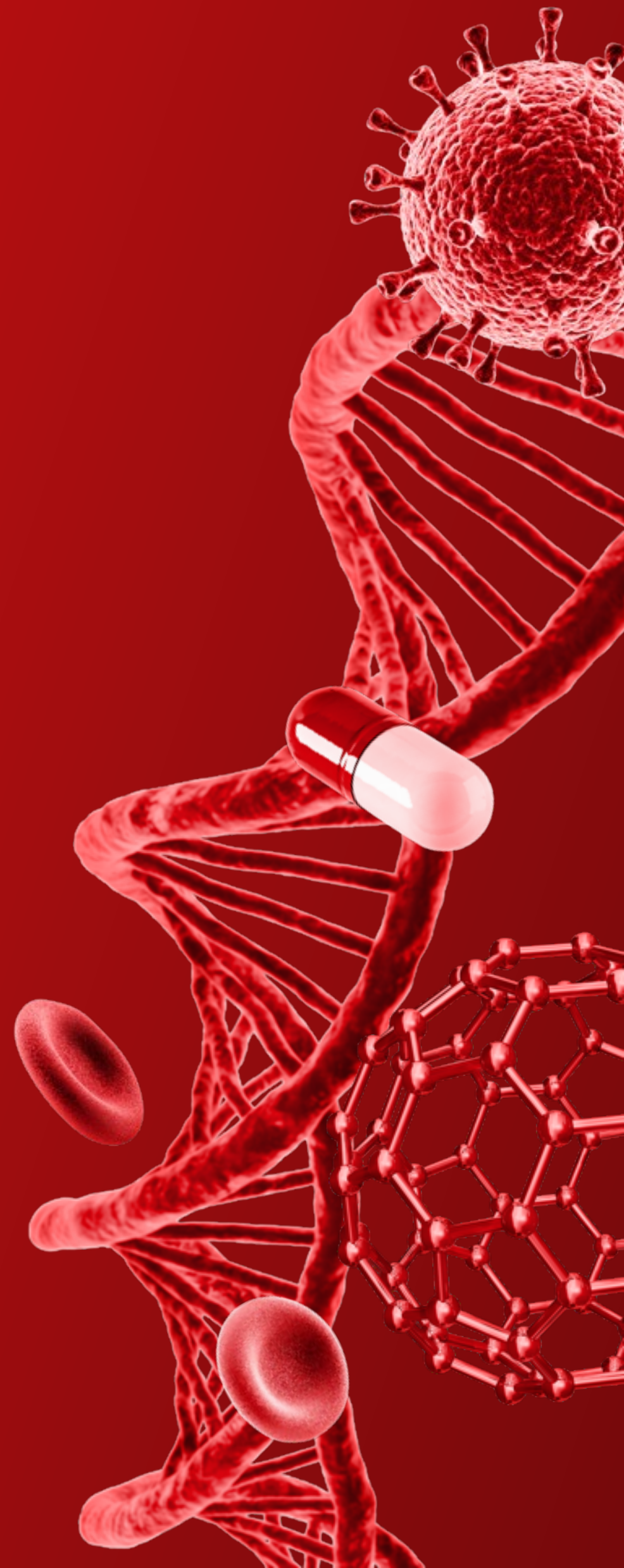

Thank you

Please submit any follow-up questions to
cellanalysis.support@thermofisher.com

For Research Use Only. Not for use in diagnostic procedures. © 2022 Thermo Fisher Scientific Inc. All rights reserved. All trademarks are the property of Thermo Fisher Scientific and its subsidiaries unless otherwise specified. OMIM is a trademark of Johns Hopkins University.
